# Current Indications and Future Direction in Heat Therapy for Musculoskeletal Pain: A Narrative Review

**DOI:** 10.3390/muscles3030019

**Published:** 2024-07-16

**Authors:** Gustavo Zanoli, Isabel Albarova-Corral, Michele Ancona, Ignazio Grattagliano, Thilo Hotfiel, Giovanni Iolascon, Karsten Krüger, Guillermo Rodríguez Maruri

**Affiliations:** 1 Orthopedic Unit, Casa di Cura Santa Maria Maddalena Rovigo, 45030 Occhiobello, Italy; 2 Department of Physiatry and Nursing, University of Zaragoza, 50009 Zaragoza, Spain; ialbarova@unizar.es; 3 Italian College of General Practitioners and Primary Care, 50142 Florence, Italy; michele.ancona@hotmail.it (M.A.); studiomedico@grattagliano.it (I.G.); 4 Center for Musculoskeletal Surgery Osnabrück (OZMC), Klinikum Osnabrück, 49076 Osnabrück, Germany; thilo.hotfiel@gmx.de; 5 Department of Medical and Surgical Specialties and Dentistry, University of Campania “Luigi Vanvitelli”, 81100 Caserta, Italy; giovanni.iolascon@unina2.it; 6 Department of Exercise Physiology and Sports Therapy, Institute of Sports Science, Justus-Liebig-University Giessen, 35390 Giessen, Germany; karsten.krueger@sport.uni-giessen.de; 7 Primary Care Musculoskeletal Unit, Area V, Servicio de Salud del Principado de Asturias, SESPA, 33203 Gijón, Spain; doctormaruri@gmail.com

**Keywords:** heat therapy, musculoskeletal pain, sports, knee pain

## Abstract

Background: Musculoskeletal pain is a non-negligible multifaceted condition affecting more than 30% of the global population. Superficial heat therapy (HT), through increasing tissue temperatures, plays a role in increasing local metabolism and function and relieving pain. Knee (KP) and sports pain represent two relevant fields of superficial HT application. Methods: In the present paper, a panel of experts performed a narrative review of the literature regarding the role of superficial HT in the management of knee and sports activity-related pain. Results: According to the reviewed literature, HT represents a therapeutic option in the management of musculoskeletal pain due to three main effects: pain relief, promotion of healing, and return to normal function and activity. Moreover, HT plays a role in sport activities both before and after exercise. Before performing sports, HT helps in preparing muscles for performance. After performing sports, it is capable to promote recovery and healing pathways. Combining and sequencing superficial heat and cold therapy represent an interesting topic of study. Overall, the application of heat wraps for superficial HT can be considered safe. Conclusions: HT has been shown to be a potentially beneficial and safe option in the management of several conditions including KP and sports. The key in the application of superficial HT is a multimodal and multidisciplinary approach.

## 1. Introduction

Musculoskeletal pain (MP) represents a non-negligible global issue affecting more than 30% of global population [1]. It causes functional disability and emotional distress, thus worsening patients’ quality of life [2]. MP can be classified into acute or chronic (i.e., lasting for more than 3 months). It can also be classified as focal (localized to a specific area) or diffuse (affecting multiple areas). There is not a single recognized cause of MP, but it may be determined either by tissue damages, sensitization, functional dysfunctions, or by undeterminable causes. Indeed, MP can occur without an identifiable structural cause, known as primary pain, or may be associated with osteo-muscular or joint diseases (secondary pain), which in turn are heterogeneously caused by inflammation and/or structural changes due to infection, crystal deposition, or auto-inflammatory pathways [2]. Despite the high social burden related to MP, it remains an underestimated problem; being such a multifaceted symptom or clinical presentation, clinicians from very different backgrounds face MP in its various forms; therefore it is not surprising that no consensus about its management has been reached so far [3]. The widespread first-line empirical treatment for MP often involves pharmacological interventions. However, recent guidelines recommend the implementation of preventative strategies, psychological therapies, and physical tools as the first option to minimize the use of medications [4]. In particular, the most recent guidelines of the American College of Rheumatology about the management of osteoarthritis of the hand, hip, and knee consider non-pharmacological treatments as effective complementary options, including heat (HT) and cold therapy [5], to facilitate a return to normal function and activity [6]. The aim of this review is to analyze current indications and future directions in HT for MP, with a focus on knee and sports pain.

## 2. Heat Therapy Overview

### 2.1. Mechanism of Action

**Figure 1:**
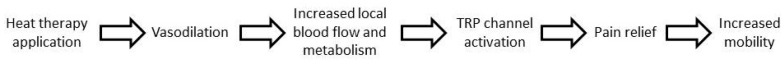
Mechanisms by which heat therapy application induces improvement in muscular pain and mobility.

HT represents one of the most ancient non-pharmacological pain treatments. It increases the temperature of a specific body area thanks to the application of an external source of heat. Some of the recognized effects of heat on tissues can be listed as follows: vasodilatation, increased blood flow, metabolism, and the function activation of the transient receptor potential (TRP) channel with pain relief [7] (Figure 1).

The application of low-level superficial heat activates temperature-sensitive nerve endings (thermoreceptors), which, in turn, initiate signals that block the processing of pain signals (nociception) in the lumbar dorsal fascia and spinal cord [8]. In addition, the pressure used to apply some superficial heat therapies, such as heat wraps, may activate the nerve endings that detect changes in tissue pressure and movement (proprioceptors); when activated, the proprioceptors block the transmission of pain signals to the spinal cord and the brain. The analgesic effects of heat are partly mediated by TRP membrane channels [9]. The TRP vanilloid 1 (TRPV1) receptors help in heat neural transduction; through their engagement, anti-nociceptive pathways in the central nervous system are activated and they cause a reduction in muscle tonicity helping their relaxation, thereby reducing MP and increasing muscle elasticity [10]. In addition, an increase in temperature tends to reduce the stiffness in fascial tissues [11].

Moreover, it is well known that an increase of 1 °C in tissue temperature can cause a 10–15% increase in the local metabolism [12]. Thus, HT plays an important role in tissue healing and regeneration by increasing catabolic and anabolic pathways, enhancing the supply of nutrients and oxygen and the removal of pain-inducing mediators produced as a by-product of tissue damage.

Kim and colleagues assessed the effects of repetitive exposure to local HT on skeletal muscle function, capillarization, myofiber morphology, and mitochondrial content. In a sample of 12 young adults, they demonstrated that local HT applied on the vastus lateralis muscle for 8 weeks promoted a proangiogenic environment and increased muscle strength, without affecting the mitochondrial content [13]. In addition, a systematic review showed preliminary evidence that repeated passive heating exposures may promote muscle hypertrophy and force improvement [14].

On the other hand, cold therapy (CT) or cryotherapy reduces blood flow to the cooled tissues, thus leading to vasoconstriction via a sympathetic reflex [6,10]. Decreasing blood flow implies the reduction of oedema and a slower delivery of inflammatory mediators to the injured area, meaning there is reduced inflammation [15,16]. Interestingly, the decreased metabolic demand in the cooled zone also prevents secondary hypoxia-related damage [17,18]. In addition, CT produces local anesthesia, namely cold-induced neurapraxia, by decreasing the activation threshold of tissue nociceptors and the conduction velocity of nerve signals conveying pain [10,19]. Finally, decreasing muscle temperature also reduces muscle spasm via the inhibition of a spinal cord reflex loop [20]. CT has been generally accepted to reduce inflammation process, perfusion, swelling, tissue damage, and pain for many years [21]. The common ways of delivering CT include cold water immersion, cold packs, cold air exposure, and ice massages. The application of CT decreases tissue temperature while reducing blood flow and microvascular perfusion through vasoconstriction and pain relief through a slowing sensory nerve conduction velocity [22].

### 2.2. Clinical Applications

HT is a widely adopted approach for MP, with evidence about its efficacy reported for both general and nonspecific conditions such as spinal (low back and neck), knee, and wrist pain and highly specific clinical conditions such as delayed onset muscle soreness (DOMS) [12,23].

For example, Petrofsky and colleagues [24] performed a randomized clinical trial to assess whether the addition at home of low-level continuous heat (i.e., commercially available heat packs to be applied 6 h before they performed their home exercise) and/or ibuprofen to physical therapy sessions could improve the outcomes of people with chronic neck pain. They demonstrated that both HT and ibuprofen were effective in reducing neck pain (VAS from around 5 to 3.5/10 in both groups, *p* < 0.05) and enhancing the neck mobility. In a recent review, including a total of 1117 adult patients, French et al. indicated that the continuous application of low-level heat directly to the skin via a heat wrap was shown to provide small, short-term improvements in pain and mobility in low back pain [25]. In particular, in adults, when compared to acetaminophen and ibuprofen, HT provides better short-term pain relief (more than 3 vs. around 2 on a 6-point scale on day 2, *p* < 0.001), significantly greater reductions in muscle stiffness, and significantly greater improvements in lateral trunk flexibility and disability scores (Roland–Morris questionnaire) [26]. The same can be said for overnight application of HT compared to placebo in adults (pain relief 2.5–3 vs. 1.5 on a 6-point scale, *p* < 0.01 or *p* < 0.001 depending on assessment time) [27]. Several studies have reported the benefits of continuous, low-level, direct heat wrap therapy for the treatment of knee pain (including pain from osteoarthritis, where a heat wrap was more effective than acetaminophen) [28,29,30,31], and wrist pain stemming from strain or sprain, tendinosis, and carpal tunnel syndrome, with particularly good results observed in the last condition (pain relief 2 vs. 1.5 on a 6-point scale, *p* < 0.05) [32]. Studies have also indicated that HT is effective at preventing and treating DOMS associated with exercise, with benefits observed in younger and older adults, as well as those with diabetes (a group who reportedly experience greater muscle soreness after exercise) [31,33,34,35].

Recently, to better address the role of HT in the treatment of MP, a panel of experts developed a list of 54 statements concerning mechanisms of action, efficacy, and the safety of HT to be disseminated through a survey to 116 European experts [36]. The panelists were asked to use a 5-point scale to agree or disagree with the proposed statements, and a threshold of 66% was set to reach the consensus according to the Delphi method. A consensus was obtained for 78% of prespecified items and the most robust were mechanism of action of heat on muscle, the indication in chronic MP, its effectiveness as part of a multimodal approach to MP, and the safety and tolerability of superficial HT [36].

### 2.3. Application Modalities

Superficial heat penetration is usually less than 1 cm, while the use of deep heat penetration is up to about 3–5 cm [37]. There are mainly three different methods through which HT can be delivered: conduction, conversion, and convection. Conduction is the transfer of heat between two objects at different temperatures through direct contact and it is the method through which hot packs, patches, wraps, and paraffin bath work. Conversion is the transfer of heat through changing from one energy form into another (i.e., ultrasound and radiant heat). Another possible way of delivering heat is convection, which consists of transferring heat by fluid circulation (liquid or gas) over the surface of a body (i.e., hydrotherapy) [12].

Many familiar ways of administering heat have been traditionally used in home settings. The prescription of applying heat might translate into different modalities in every household, depending also on cultural habits and geographic location. Our panel underlined that standardizing heat administration with commercially available devices, while “medicalizing” a simple therapeutic approach, could also help compliance and the scientific evaluation of the effectiveness of HT in clinical settings.

### 2.4. Safety

The cited Delphi consensus also provided indications on safety concerns regarding HT based on heat wraps [36]. According to the results, caution should be executed in patients with active immune diseases, cancer, active osteoarthritis, neurological diseases, zoster infections, skin injuries/conditions, and circulation defects. Moreover, skin integrity represents a non-negligible criterion in selecting potential candidates for HT [36].

Reassuringly, no deaths and no serious adverse events have been reported so far for superficial HT based on heat wraps. Mild heat-related skin adverse events were reported including redness/pinkness and first/second degree burns. All the skin effects related to thermal injury were infrequent, mild-to-moderate, and independently recovered [27]. Overall, the application of heat wraps for superficial HT can be considered safe. During the discussion, some members of the panel hypothesized that, after taking into account possible allergies to other components (e.g., glue in adhesive patches), standardized devices could help reduce heat-related local adverse events, especially in less diligent patients.

### 2.5. Heat Therapy versus Cold Therapy, Which and When

Clinical members of the panel reported a frequent question posed by patients regarding the relative role of heat and cold therapy (CT) in dealing with their MP. Therefore, some considerations resulting from the literature review will be recapped hereafter.

When choosing between HT and CT, the phases of the healing process including the inflammatory phase, the proliferation phase, and the remodelling phase should be considered. The first phase can last for 1 to 3 days, varying based on injury severity, and is determined by a pro-inflammatory mechanism to clean up the damaged area as well as to create the basis for the cellular process of regeneration [38]. During this phase, CT can help in limiting inflammatory process and pain. On the contrary, HT may exacerbate inflammation and oedema through enhanced vasodilation [36]. During the second phase, new tissue and scar tissue are formed based on cell activation, angiogenesis, and extracellular matrix (ECM) formation. Heat can be applied to the injured area to facilitate the healing process [10,39]. The third phase is the process of returning to health: the restoration of the structure and function of injured or diseased tissues. In this phase, the blood supply is restored by the release of angiogenic factors, together with the restoration of structural tissue integrity, the extracellular matrix, and the neuronal innervation of the injured muscle fibres. Here, HT can improve the release of myogenic growth factors [40].

As a broad guideline, CT is mainly applied in acute and/or traumatic conditions and in case of chronic damage with predominant active inflammation (i.e., during the initial 48 to 72 h after an acute injury) [41], while HT should be applied once the inflammatory phase is recovered [42].

Interestingly, chronic pain is also related to complex regional pain syndrome (CRPS), which shows involvement of the vascular system, particularly microvasculature, and triggers inflammation to sustain its pathophysiology [43]. The syndrome usually follows deep-tissue injuries such as fractures, crush injuries, or sprains. Generally, CRPS is characterized by a short initial “hot” phase (weeks–months) with increased skin temperature, oedema, sweating, and inflammation. This is followed by a “mixed hot/cold” phase, and finally by a chronic “cold” phase (years), which shows lowered skin temperature, dryness, and cyanosis [43]. We can hypothesize that in this clinical scenario, CT should also be applied in the “hot” phase, and HT is useful in the long “cold” phase.

Nevertheless, most recommendations for the application in clinical practice of HT and CT are based on empirical experience and “good clinical practice” and there is a lack of powered, high-quality randomized clinical trials. In addition, many patients apply superficial HT or CT irrespective of scientific rationale or healthcare professional advice, so it is crucial to better address the correct indications and contraindications of these two methods.

### 2.6. Specific Applications of Heat Therapy

After this general overview of the basic notions and clinical uses of HT, the rest of this paper will be aimed at narratively reviewing the literature regarding potential HT applications, focusing specifically on knee and sports activity-related pain, as it represents the expertise area of the authors.

## 3. Heat Therapy and Knee Pain

### 3.1. Knee Pain as a Multifaceted Clinical Condition

Knee pain (KP) is a common clinical condition related with trauma, overuse, or degenerative injuries with a range of presentations that varies from acute to chronic pain. Among possible anatomic structures causing KP, there are intra-articular structures, and surrounding tendons, bones, ligaments, and muscles [44]. Joint pain is the most common joint symptom in the world, with an age-related increase in both incidence and prevalence [45].

Knee injuries are very frequent among athletes [46] and the general population [47], and inevitably are a major source of possible KP. Non-traumatic KP is mainly caused by osteoarthritis (OA) and results from the breakdown of the articular cartilage, causing stiffness, pain, redness, heat, joint swelling, and a decreased range of motion (ROM); the knee is one of the most frequently involved joints in OA. Typically, pain or stiffness in the joint occurs after periods of inactivity (inflammatory pain) or excessive use (mechanical pain) [48]. Symptoms of OA usually progress slowly over a period lasting years, occurring initially after exercise and becoming constant over time, also interfering with normal daily activities and the patient’s quality of life (QoL) [48].

Tendon and ligament dysfunction can also be responsible for KP occurrence. Overload, disrepair, and structural damages of these structures are the most common causes of pain [49].

A myofascial trigger point is a hyperirritable spot, usually within a taut band of skeletal muscle, which is painful on compression and can give rise to characteristic referred pain in a specific and reproducible area, motor dysfunction, and autonomic phenomena [50]. Thus, the origin of KP can also be the muscle and the trigger point of related KP has been widely described [51].

Other common causes of KP include patellofemoral pain syndrome (PFPS) and Iliotibial band syndrome (ITBS), which cause anterior or lateral KP, respectively. Typical symptoms include pain behind or around the patella or on the lateral border of the knee that increases with running and activities that involve knee flexion, and pain may worsen after a period of rest [52,53,54].

### 3.2. Heat Therapy for the Management of Knee Pain

Both HT and CT are recommended as non-invasive and non-pharmacological treatments for MP by different international guidelines, including the Canadian and American College of Rheumatology guides [55,56]. Other institutions choose not to include thermal modalities in their recommendations since they were not considered “core” treatments [57].

Petrofsky and colleagues [28] performed a randomized controlled trial to assess whether the use of a low level of continuous heat (LLCH) can improve KP recovery when added to physical therapy in patients with chronic KP. Fifty patients were randomly allocated to experimental or placebo arms of the study. All enrolled patients received both conventional physical therapies, and they were asked to perform therapeutic exercises at home each day between sessions. In adjunction, patients allocated in the experimental arm applied LLCH knee wraps for 6 h at home before home exercises. The LLCH group showed a significant pain attenuation compared to the placebo group (VAS from 4.1 to 1.6/10 vs. 4.6 to 3.5/10, respectively, *p* < 0.05), and patients in the experimental arm showed a significantly higher rate of home exercise compliance.

Kim and colleagues [58] evaluated, in a randomized controlled trial, the effects of adding HT to exercise in the management of elderly women’s chronic KP. One hundred and fifty women over 75 years old complaining of KP were randomly allocated to receive either physical therapy plus HT, physical therapy alone, HT alone, or health education. The results showed a visual analogue scale (VAS) improvement in patients treated with HT, both alone (from 4.6 to 3.4/10) and combined with exercise (from 3.6 to 2.0/10). The total Japanese Knee Osteoarthritis Measure (JKOM) score, muscle strength, and functional mobility significantly improved in the group of patients that received the combination of HT and physical therapy. According to the study results, when combined with exercise HT seems to help in reducing pain, improving physical function, and increasing the patient’s quality of life.

The effects of HT in the management of KP were investigated in another clinical trial conducted on eighteen women aged between 50 and 69 years old. Patients were randomly assigned to a 12-week intervention with HT alone or exercise alone, and the resulting effects were evaluated using magnetic resonance imaging (MRI) T2 mapping. Local HT applied with a standardized method and evaluated after 12 weeks improved patients’ clinical scores (Japanese Knee Osteoarthritis Measure) and MRI measurements of cartilage thickness [59].

### 3.3. Heat versus Cold Therapy in Knee Pain

So far, there is only sparse evidence in the literature regarding the use of either HT or CT in the management of KP. Therefore, it is crucial to highlight the role of these two different approaches and define the correct sequence of application.

In a trial comparing the effect of HT and CT on pain and joint function in knee OA, 117 patients were randomly allocated to receive CT, HT, or placebo control. Both CT and HT proved to be significantly effective in reducing pain and improving joint function when compared to the placebo [60].

Aciksoz and colleagues [61], in a randomized controlled trial, investigated the effect of superficial cold and heat applications on pain, functional status, and quality of life in 96 cases of knee OA. In the two experimental groups, patients received, on top of standard orthopaedic treatment, either superficial HT or CT. Both therapies were applied for 20 min 2 times a day, in the morning and in the evening, for 3 weeks. Both heat and cold superficial applications determined a mild improvement in pain (less than 1/10 in VAS, *p* < 0.01), functional status, and quality of life. However, no significant differences were found between the two interventional groups.

Moreover, the possible treatment sequence of applying HT followed by CT to improve passive knee flexion was evaluated in subjects with restricted knee motion. Seventy-one subjects were randomized to receive either HT–CT sequential treatment or HT alone. Interestingly, statistically limited but significant increments of knee flexion were observed for subjects receiving the sequential approach [62].

## 4. Heat Therapy and Sport

Performing sports is one of the main recommendations for a healthy lifestyle both in children [63] and in the elderly [64,65]. Although the health benefits of sports normally outweigh the risks, it is estimated that one out of three working adults lost at least one day of work a year due to a sport-related injury [66,67].

Warming up by increasing the tissue temperature is recognized as an effective method to increase tissue distensibility and reduce the incidence of injury [68,69,70]. Among possible warm-up modalities are active treatments such as exercise to increase circulation and metabolism, myofascial treatments such as stretching or foam rolling, passive interventions such as heat application or massage, or even mental conditioning [69,70,71,72].

However, it remains unclear which factors can reduce the risk of sports-related injuries, and which can be the best method of prevention management.

### 4.1. Heat Therapy Application in Sport

Empirically, HT is very commonly used before exercise. In addition, basic scientific studies show that the increased tissues metabolism due to local heating helps in preparing muscles for athletic performance [29,68,73]. Moreover, HT is capable of promoting recovery after exercise thanks to the possibly increased removal of metabolism products, which increases the delivery of nutrients and glycogen resynthesis and promotes healing pathways [74,75].

One of the most interesting applications of superficial HT in sport is represented by the increased joint flexibility after heat application. This effect of HT can play a crucial role in reducing both the chance of injury and the energy cost of muscle contraction [29].

Some in vivo studies on animal models provided insights on the rationale of using HT in sport activities. In rat tendon models, increasing tissue temperature and then maintaining it before applying force, significantly less damage occurred. Also, at elevated temperatures the lower loads applied for prolonged periods produced significantly greater residual elongation [76].

A trial performed on twenty-two female collegiate lacrosse and soccer athletes showed that HT improved the hip ROM. Interestingly, the trial highlighted how athlete perception is not always reliable and professionals should evaluate and prescribe treatment on a case-by-case basis [77].

In a systematic review on the effects of stretching with or without HT on ROM, twelve studies including 352 participants were retrieved. The subgroup analyses demonstrated a greater improved ROM after heat combined with stretching, compared to HT alone. However, a subgroup analysis of muscle groups and different HT-delivering methods did not show significant differences [78].

It remains unclear whether superficial HT can be used at different times during sporting activity, such as during the performance to enhance it. Future research should confirm these observations.

### 4.2. Heat versus Cold Therapy in Sport

As mentioned above for KP management, the possibility of combining, sequencing, or switching HT and CT in sport represents an interesting and debated topic.

Twenty subjects of both sexes were enrolled in a trial designed to determine if HT would increase the extensibility of the anterior and posterior knee cruciate ligaments and reduce the force needed to flex the joint. Results proved that HT increased ligaments’ flexibility and the force needed to flex the knee was reduced by 25% when compared to CT [70].

In a systematic review and meta-analysis of 32 randomized controlled trials on the effects of HT and CT, it was proven that CT could reduce DOMS within 1 h after exercise. Additionally, HT could reduce the pain of patients within and in periods over 24 h [23].

Moreover, a network metanalysis reported that within 24 and 48 h postexercise, hot pack application was superior to other interventions, whereas over 48 h, CT was the optimal intervention for pain relief in patients with DOMS. Authors underlined that HT had a better safety and convenience profile [79].

## 5. Discussion

MP represents a global health issue with medical implications Patients with MP are seen in many different specialized settings, but very often in general medicine and even by different healthcare professionals. Despite its frequency, the study of its prevention and treatment is complicated by the fact that it does not recognize a specific and unique aetiology [1,2,3].

Reviewing the basic science presented, the expert panel agreed that superficial HT—through increasing tissues’ temperature—can enhance local metabolism and function and thus relieve pain [7,11].

The evidence gathered for this narrative review, though sparse and partial in the opinion of the experts involved, showed HT to be a potentially useful, safe, and cost-effective tool in many types of MP [11,23,24,25,26,27,28,29,30,31,32,33,34,35], specifically in knee and sports activity-related MP, which represent two of the most intriguing fields of application and research for the use of superficial HT. The novelty of this review is, indeed, a complete focus on knee and sports pain with regards to the usefulness of HT provided by experts in this field.

HT alone or in combination with other treatments has been found to be beneficial in the management of KP conditions in three domains: pain relief, promotion of healing, and a return to normal function and activity [28,58,59,60,61,62]. On the other hand, HT can play a role in sport both before and after exercise [29,68,70,73,74,75,76,77,78,79]. Before performing sports, HT might help in preparing muscles for athletic performance. After performing physical activity, it could promote recovery and healing pathways. Both methods of HT delivery may have implications in injury prevention and pain reduction.

Despite being an ancient therapeutical approach and therefore often regarded rather as a self-management strategy, it is the opinion of this panel that HT deserves attention in clinical research and practice guidelines. Several gray areas should be covered in future studies. Until now, there have been no clear cut and evidence-based recommendations in the application of HT. Randomized controlled trials are needed investigating the long-term clinical outcomes in patients who were allocated to different and clearly defined thermotherapies (HT and CT) with different indications. Further, the role of HT as a part of multimodal and multidisciplinary approaches should be investigated. Understanding how HT can be integrated into comprehensive treatment plans could improve patient outcomes. With technological advancements, innovative methods for delivering HT (e.g., diathermy, tecartherapy, or wearable heat patches/devices) are being developed. However, studies evaluating how effective, safe, and user-friendly these new technologies are, remain limited.

In general, most of the existing scientific studies and research focus on the mechanisms and benefits of HT. To date, there is a lack of knowledge in regard to potential harms and side effects. Even if HT has been considered as a safe method, potential risks and side effects have to be investigated in more detail. 

Future research should be aimed both at investigating the role of HT in preventing or treating MP as a clinical symptom in a general setting, and as a part of more specific underlying diagnoses in the different medical specialties involved. Randomized controlled trials to evaluate the efficacy and safety of HT in different populations should use appropriate validated outcome measures, and could have the benefit of providing HT through standardized methods, which should improve repeatability, compliance, safety, and external validity. Practicing clinicians and guidelines should consider HT.

To conclude, HT is a widely used tool to treat nonspecific MP. However, specific indications can be advised regarding KP and sports pain, typically favoring pain relief and promoting healing and a return to normal function and activity. In this context, a pathophysiology-guided approach is useful to alternate CT and HT according to the clinical phase of the damage. In addition, HT can also be adopted before exercise to prepare muscles for athletic performance, and after physical activity to promote recovery and healing pathways. Several gray areas, namely specific indications/pathologies, therapy application specifications (location, time, etc), combined therapies strategies results, different modalities of HT, and side effects/safety concerns, should be explored in future research and randomized trials.

## 6. Materials and Methods

An expert panel of clinicians and research experts in KP, representing different medical specialties and from several European countries, was gathered to discuss the potential role of superficial HT in conditions that present with some degree of MP. Nine experts on MP, including one orthopaedic surgeon, one sports medicine physician, two family medicine physicians, one physiotherapist, two physiatrists, and one exercise physiologist were selected based on their previous experience in HT.

The experts were encouraged to express their opinion on MP management and the role of HT in this context, particularly focusing on KP. After one virtual meeting (where a note taker and an audio registration recorded every opinion) and both personal and centralized literature reviews, the expert panel decided to produce a narrative review to summarize the knowledge regarding the application of HT in preventing and treating MP, with a special focus on KP and sport activities. 

Only opinions shared unanimously are reported in this paper. The results of the information collected, and the opinions expressed have been summarized in this paper, starting from a general overview of what is known about HT and its possible mechanism of action, its clinical application and safety issues, its role in comparison or in combination with CT, and then proceeding by summarizing the evidence related to KP and sport activities. This study, as a literature review, is exempt from Institutional Review Board approval. One member of the panel decided (for personal and administrative reasons) not to appear as an author of this paper. Every other panelist completely revised and approved this paper.

## Data Availability

No new data were created in this study.

## References

[1] GBD 2019 Diseases and Injuries Collaborators (2019). Global burden of 369 diseases and injuries in 204 countries and territories, 1990–2019: A systematic analysis for the Global Burden of Disease Study. Lancet.

[2] Puntillo F., Giglio M., Paladini A., Perchiazzi G., Viswanath O., Urits I., Sabbà C., Varrassi G., Brienza N. (2021). Pathophysiology of musculoskeletal pain: A narrative review. Ther. Adv. Musculoskelet. Dis..

[3] Blyth F.M., Briggs A.M., Schneider C.H., Hoy D.G., March L.M. (2019). The Global Burden of Musculoskeletal Pain—Where to From Here?. Am. J. Public Health.

[4] El-Tallawy S.N., Nalamasu R., Salem G.I., LeQuang J.A.K., Pergolizzi J.V., Christo P.J. (2021). Management of Musculoskeletal Pain: An Update with Emphasis on Chronic Musculoskeletal Pain. Pain. Ther..

[5] Kolasinski S.L., Neogi T., Hochberg M.C., Oatis C., Guyatt G., Block J., Callahan L., Copenhaver C., Dodge C., Felson D. (2020). 2019 American College of Rheumatology/Arthritis Foundation Guideline for the Management of Osteoarthritis of the Hand, Hip, and Knee. Arthritis Rheumatol..

[6] Malanga G.A., Yan N., Stark J. (2015). Mechanisms and efficacy of heat and cold therapies for musculoskeletal injury. Postgrad. Med..

[7] Papaioannou T.G., Karamanou M., Protogerou A.D., Tousoulis D. (2016). Heat therapy: An ancient concept re-examined in the era of advanced biomedical technologies. J. Physiol..

[8] Green B.G. (2004). Temperature perception and nociception. J. Neurobiol..

[9] Brederson J.D., Kym P.R., Szallasi A. (2013). Targeting TRP channels for pain relief. Eur. J. Pharmacol..

[10] Nadler S.F., Weingand K., Kruse R.J. (2004). The physiologic basis and clinical applications of cryotherapy and thermotherapy for the pain practitioner. Pain. Physician.

[11] Klingler W., Schleip R., Findley T.W., Chaitow L., Huijing P. (2012). Temperature effects on fascia. Fascia—The Tensional Network of the Human Body.

[12] Freiwald J., Magni A., Fanlo-Mazas P., Paulino E., Sequeira de Medeiros L., Moretti B., Schleip R., Solarino G. (2021). A Role for Superficial Heat Therapy in the Management of Non-Specific, Mild-to-Moderate Low Back Pain in Current Clinical Practice: A Narrative Review. Life.

[13] Kim K., Reid B.A., Casey C.A., Bender B.E., Ro B., Song Q., Trewin A.J., Petersen A.C., Kuang S., Gavin T.P. (2020). Effects of repeated local heat therapy on skeletal muscle structure and function in humans. J. Appl. Physiol..

[14] Rodrigues P., Trajano G.S., Wharton L., Minett G.M. (2020). Effects of passive heating intervention on muscle hypertrophy and neuromuscular function: A preliminary systematic review with meta-analysis. J. Therm. Biol..

[15] Deal D.N., Tipton J., Rosencrance E., Curl W.W., Smith T.L. (2002). Ice reduces edema. A study of microvascular permeability in rats. J. Bone Jt. Surg. Am..

[16] Schaser K.D., Vollmar B., Menger M.D., Schewior L., Kroppenstedt S.N., Raschke M., Lübbe A.S., Haas N.P., Mittlmeier T. (1999). In vivo analysis of microcirculation following closed soft-tissue injury. J. Orthop. Res..

[17] Merrick M.A., Rankin J.M., Andres F.A., Hinman C.L. (1999). A preliminary examination of cryotherapy and secondary injury in skeletal muscle. Med. Sci. Sports Exerc..

[18] Sapega A.A., Heppenstall R.B., Sokolow D.P., Graham T.J., Maris J.M., Ghosh A.K., Chance B., Osterman A.L. (1988). The bioenergetics of preservation of limbs before replantation. The rationale for intermediate hypothermia. J. Bone Jt. Surg. Am..

[19] Algafly A.A., George K.P. (2007). The effect of cryotherapy on nerve conduction velocity, pain threshold and pain tolerance. Br. J. Sports Med..

[20] Lee S.U., Bang M.S., Han T.R. (2002). Effect of cold air therapy in relieving spasticity: Applied to spinalized rabbits. Spinal Cord..

[21] Allan R., Malone J., Alexander J., Vorajee S., Ihsan M., Gregson W., Kwiecien S., Mawhinney C. (2022). Cold for centuries: A brief history of cryotherapies to improve health, injury and post-exercise recovery. Eur. J. Appl. Physiol..

[22] Kwiecien S.Y., McHugh M.P. (2021). The cold truth: The role of cryotherapy in the treatment of injury and recovery from exercise. Eur. J. Appl. Physiol..

[23] Wang Y., Li S., Zhang Y., Chen Y., Yan F., Han L., Ma Y. (2021). Heat and cold therapy reduce pain in patients with delayed onset muscle soreness: A systematic review and meta-analysis of 32 randomized controlled trials. Phys. Ther. Sport.

[24] Petrofsky J.S., Laymon M., Alshammari F., Khowailed I.A., Lee H. (2017). Use of low level of continuous heat and Ibuprofen as an adjunct to physical therapy improves pain relief, range of motion and the compliance for home exercise in patients with nonspecific neck pain: A randomized controlled trial. J. Back. Musculoskelet. Rehabil..

[25] French S.D., Cameron M., Walker B.F., Reggars J.W., Esterman A.J. (2006). Superficial heat or cold for low back pain. Cochrane Database Syst. Rev..

[26] Nadler S.F., Steiner D.J., Erasala G.N., Hengehold D.A., Hinkle R.T., Beth Goodale M., Abeln S.B., Weingand K.W. (2002). Continuous low-level heat wrap therapy provides more efficacy than Ibuprofen and acetaminophen for acute low back pain. Spine (Phila Pa 1976).

[27] Nadler S.F., Steiner D.J., Erasala G.N., Hengehold D.A., Abeln S.B., Weingand K.W. (2003). Continuous low-level heatwrap therapy for treating acute nonspecific low back pain. Arch. Phys. Med. Rehabil..

[28] Petrofsky J.S., Laymon M.S., Alshammari F.S., Lee H. (2016). Use of Low Level of Continuous Heat as an Adjunct to Physical Therapy Improves Knee Pain Recovery and the Compliance for Home Exercise in Patients with Chronic Knee Pain: A Randomized Controlled Trial. J. Strength Cond. Res..

[29] Petrofsky J., Laymon M., Alshammari F., Khowailed I.A., Lee H. (2015). Continuous Low Level Heat Wraps; Faster Healing and Pain Relief during Rehabilitation for Back, Knee and Neck Injuries. World J. Prev. Med..

[30] McCarberg W., Erasala G., Goodale M., Grender J.M., Hengehold D., Donikyan L. (2005). Therapeutic benefits of Continuous Low-level Heat wrap Therapy (CLHT) for Osteoarthritis (OA) of the knee. J. Pain..

[31] Draper D.O., Hopkins T.J. (2008). Increased intramuscular and intracapsular temperature via ThermaCare Knee Wrap application. Med. Sci. Monit..

[32] Michlovitz S., Hun L., Erasala G.N., Hengehold D.A., Weingand K.W. (2004). Continuous low-level heat wrap therapy is effective for treating wrist pain. Arch. Phys. Med. Rehabil..

[33] Mayer J.M., Mooney V., Matheson L.N., Erasala G.N., Verna J.L., Udermann B.E., Leggett S. (2006). Continuous low-level heat wrap therapy for the prevention and early phase treatment of delayed-onset muscle soreness of the low back: A randomized controlled trial. Arch. Phys. Med. Rehabil..

[34] Petrofsky J., Batt J., Bollinger J.N., Jensen M.C., Maru E.H., Al-Nakhli H.H. (2011). Comparison of different heat modalities for treating delayed onset muscle soreness in people with diabetes. Diabetes Technol. Ther..

[35] Heiss R., Lutter C., Freiwald J., Hoppe M.W., Grim C., Poettgen K., Forst R., Bloch W., Hüttel M., Hotfiel T. (2019). Advances in Delayed-Onset Muscle Soreness (DOMS)—Part II: Treatment and Prevention. Sportverletz. Sportschaden.

[36] Lubrano E., Mazas P.F., Freiwald J., Krüger K., Grattagliano I., Mur E., Silva R.Q., Maruri G.R., de Medeiros L.S. (2023). An International Multidisciplinary Delphi-Based Consensus on Heat Therapy in Musculoskeletal Pain. Pain. Ther..

[37] Chen W.S., Annaswamy T.M., Yang W., Wang T.G., Kwon D.R., Chou L.W. (2021). Physical Agent Modalities. Braddom’s Physical Medicine and Rehabilitation.

[38] Forcina L., Cosentino M., Musarò A. (2020). Mechanisms Regulating Muscle Regeneration: Insights into the Interrelated and Time-Dependent Phases of Tissue Healing. Cells.

[39] Lohman E.B., Sackiriyas K.S., Bains G.S., Calandra G., Lobo C., Nakhro D., Malthankar G., Paul S. (2012). A comparison of whole body vibration and moist heat on lower extremity skin temperature and skin blood flow in healthy older individuals. Med. Sci. Monit..

[40] Kim W.S., Kim J. (2023). Exploring the impact of temporal heat stress on skeletal muscle hypertrophy in bovine myocytes. J. Therm. Biol..

[41] Sluka K.A., Christy M.R., Peterson W.L., Rudd S.L., Troy S.M. (1999). Reduction of pain-related behaviors with either cold or heat treatment in an animal model of acute arthritis. Arch. Phys. Med. Rehabil..

[42] Hotfiel T., Hoppe M.W., Heiss R., Lutter C., Tischer T., Forst R., Hammer C.M., Freiwald J., Engelhardt M., Grim C. (2021). Quantifiable Contrast-Enhanced Ultrasound Explores the Role of Protection, Rest, Ice (Cryotherapy), Compression and Elevation (PRICE) Therapy on Microvascular Blood Flow. Ultrasound Med. Biol..

[43] Coderre T.J. (2023). Contribution of microvascular dysfunction to chronic pain. Front. Pain Res..

[44] Hadler N.M. (1992). Knee pain is the malady—Not osteoarthritis. Ann. Intern. Med..

[45] Mandl L.A. (2019). Osteoarthritis year in review 2018: Clinical. Osteoarthr. Cartil..

[46] Kujala U.M., Taimela S., Antti-Poika I., Orava S., Tuominen R., Myllynen P. (1995). Acute injuries in soccer, ice hockey, volleyball, basketball, judo, and karate: Analysis of national registry data. BMJ.

[47] Gage B.E., McIlvain N.M., Collins C.L., Fields S.K., Comstock R.D. (2012). Epidemiology of 6.6 Million Knee Injuries Presenting to United States Emergency Departments from 1999 Through 2008. Acad. Emerg. Med..

[48] Hunter D.J., McDougall J.J., Keefe F.J. (2008). The Symptoms of Osteoarthritis and the Genesis of Pain. Rheum. Dis. Clin. N. Am..

[49] Madi S., Acharya K., Pandey V. (2022). Current concepts on management of medial and posteromedial knee injuries. J. Clin. Orthop. Trauma..

[50] Lavelle E.D., Lavelle W., Smith H.S. (2007). Myofascial trigger points. Anesthesiol. Clin..

[51] Rahou-El-Bachiri Y., Navarro-Santana M.J., Gómez-Chiguano G.F. (2020). Effects of Trigger Point Dry Needling for the Management of Knee Pain Syndromes: A Systematic Review and Meta-Analysis. J. Clin. Med..

[52] Gaitonde D.Y., Ericksen A., Robbins R.C. (2019). Patellofemoral Pain Syndrome. Am. Fam. Physician.

[53] D’Ambrosi R., Meena A., Raj A., Ursino N., Hewett T.E. (2022). Anterior Knee Pain: State of the Art. Sport Med.—Open.

[54] Strauss E.J., Kim S., Calcei J.G., Park D. (2011). Iliotibial band syndrome: Evaluation and management. J. Am. Acad. Orthop. Surg..

[55] Qaseem A., Wilt T.J., McLean R.M., Forciea M.A. (2017). Noninvasive Treatments for Acute, Subacute, and Chronic Low Back Pain: A Clinical Practice Guideline From the American College of Physicians. Ann. Intern. Med..

[56] Hochberg M.C., Altman R.D., April K.T., Benkhalti M., Guyatt G., McGowan J., Towheed T., Welch V., Wells G., Tugwell P. (2012). American College of Rheumatology 2012 recommendations for the use of nonpharmacologic and pharmacologic therapies in osteoarthritis of the hand, hip, and knee. Arthritis Care Res..

[57] Fernandes L., Hagen K.B., Bijlsma J.W., Andreassen O., Christensen P., Conaghan P.G., Doherty M., Geenen R., Hammond A., Kjeken I. (2013). EULAR recommendations for the non-pharmacological core management of hip and knee osteoarthritis. Ann. Rheum. Dis..

[58] Kim H., Suzuki T., Saito K., Kim M., Kojima N., Ishizaki T., Yamashiro Y., Hosoi E., Yoshida H. (2013). Effectiveness of exercise with or without thermal therapy for community-dwelling elderly Japanese women with non-specific knee pain: A randomized controlled trial. Arch. Gerontol. Geriatr..

[59] Ochiai S., Watanabe A., Oda H., Ikeda H. (2014). Effectiveness of Thermotherapy Using a Heat and Steam Generating Sheet for Cartilage in Knee Osteoarthritis. J. Phys. Ther. Sci..

[60] Ariana M., Afrasiabifar A., Najafi Doulatabad S., Mosavi A., Behnammoghadam M. (2022). The Effect of Local Heat Therapy versus Cold Rub Gel on Pain and Joint Functions in Patients with Knee Osteoarthritis. Clin. Nurs. Res..

[61] Aciksoz S., Akyuz A., Tunay S. (2017). The effect of self-administered superficial local hot and cold application methods on pain, functional status and quality of life in primary knee osteoarthritis patients. J. Clin. Nurs..

[62] Lin Y.H. (2003). Effects of thermal therapy in improving the passive range of knee motion: Comparison of cold and superficial heat applications. Clin. Rehabil..

[63] Fathi Azar E., Mirzaie H., Jamshidian E., Hojati E. (2023). Effectiveness of perceptual-motor exercises and physical activity on the cognitive, motor, and academic skills of children with learning disorders: A systematic review. Child. Care Health Dev..

[64] Sharifi M., Nodehi D., Bazgir B. (2023). Physical activity and psychological adjustment among retirees: A systematic review. BMC Public Health.

[65] Malm C., Jakobsson J., Isaksson A. (2019). Physical Activity and Sports-Real Health Benefits: A Review with Insight into the Public Health of Sweden. Sports.

[66] Conn J.M. (2003). Sports and recreation related injury episodes in the US population, 1997–1999. Inj. Prev..

[67] Emery C.A., Pasanen K. (2019). Current trends in sport injury prevention. Best Pract. Res. Clin. Rheumatol..

[68] Pasanen K., Parkkari J., Pasanen M., Kannus P. (2009). Effect of a neuromuscular warm-up programme on muscle power, balance, speed and agility: A randomised controlled study. Br. J. Sports Med..

[69] LaBella C.R., Huxford M.R., Grissom J., Kim K.Y., Peng J., Christoffel K.K. (2011). Effect of Neuromuscular Warm-up on Injuries in Female Soccer and Basketball Athletes in Urban Public High Schools. Arch. Pediatr. Adolesc. Med..

[70] Petrofsky J.S., Laymon M., Lee H. (2013). Effect of heat and cold on tendon flexibility and force to flex the human knee. Med. Sci. Monit..

[71] McGowan C.J., Pyne D.B., Thompson K.G., Rattray B. (2015). Warm-Up Strategies for Sport and Exercise: Mechanisms and Applications. Sports Med..

[72] Bishop D. (2003). Warm up I: Potential mechanisms and the effects of passive warm up on exercise performance. Sports Med..

[73] Kim K., Monroe J.C., Gavin T.P., Roseguini B.T. (2020). Local Heat Therapy to Accelerate Recovery After Exercise-Induced Muscle Damage. Exerc. Sport Sci. Rev..

[74] Heinonen I., Brothers R.M., Kemppainen J., Knuuti J., Kalliokoski K.K., Crandall C.G. (2011). Local heating, but not indirect whole body heating, increases human skeletal muscle blood flow. J. Appl. Physiol..

[75] Fradkin A.J., Zazryn T.R., Smoliga J.M. (2010). Effects of warming-up on physical performance: A systematic review with meta-analysis. J. Strength. Cond. Res..

[76] Warren C.G., Lehmann J.F., Koblanski J.N. (1976). Heat and stretch procedures: An evaluation using rat tail tendon. Arch. Phys. Med. Rehabil..

[77] Oranchuk D.J., Flattery M.R., Robinson T.L. (2019). Superficial heat administration and foam rolling increase hamstring flexibility acutely; with amplifying effects. Phys. Ther. Sport.

[78] Nakano J., Yamabayashi C., Scott A., Reid W.D. (2012). The effect of heat applied with stretch to increase range of motion: A systematic review. Phys. Ther. Sport.

[79] Wang Y., Lu H., Li S., Zhang Y., Yan F., Huang Y., Chen X., Yang A., Han L., Ma Y. (2022). Effect of cold and heat therapies on pain relief in patients with delayed onset muscle soreness: A network meta-analysis. J. Rehabil. Med..

